# Age-Related Maintenance of the Autophagy-Lysosomal System Is Dependent on Skeletal Muscle Type

**DOI:** 10.1155/2020/4908162

**Published:** 2020-07-24

**Authors:** Raquel Fernando, José Pedro Castro, Tanina Flore, Stefanie Deubel, Tilman Grune, Christiane Ott

**Affiliations:** ^1^Department of Molecular Toxicology, German Institute of Human Nutrition Potsdam-Rehbrücke, 14558 Nuthetal, Germany; ^2^Division of Genetics, Brigham and Women's Hospital and Harvard Medical School, Boston, MA 02115, USA; ^3^German Center for Diabetes Research (DZD), 85764 München-Neuherberg, Germany; ^4^University of Potsdam, Institute of Nutritional Science, 14558 Nuthetal, Germany; ^5^German Center for Cardiovascular Research (DZHK), 10117 Berlin, Germany

## Abstract

The skeletal muscle plays an important role in maintaining whole-body mechanics, metabolic homeostasis, and interorgan crosstalk. However, during aging, functional and structural changes such as fiber integrity loss and atrophy can occur across different species. A commonly observed hallmark of aged skeletal muscle is the accumulation of oxidatively modified proteins and protein aggregates which point to an imbalance in proteostasis systems such as degradation machineries. Recently, we showed that the ubiquitin-proteasomal system was impaired. Specifically, the proteasomal activity, which was declining in aged *M. soleus* (SOL) and *M. extensor digitorum longus* (EDL). Therefore, in order to understand whether another proteolytic system would compensate the decline in proteasomal activity, we aimed to investigate age-related changes in the autophagy-lysosomal system (ALS) in SOL, mostly consisting of slow-twitch fibers, and EDL, mainly composed of fast-twitch fibers, from young (4 months) and old (25 months) C57BL/6JRj mice. Here, we focused on changes in the content of modified proteins and the ALS. Our results show that aged SOL and EDL display high levels of protein modifications, particularly in old SOL. While autophagy machinery appears to be functional, lysosomal activity declines gradually in aged SOL. In contrast, in old EDL, the ALS seems to be affected, demonstrated by an increased level of key autophagy-related proteins, which are known to accumulate when their delivery or degradation is impaired. In fact, lysosomal activity was significantly decreased in old EDL. Results presented herein suggest that the ALS can compensate the high levels of modified proteins in the more oxidative muscle, SOL, while EDL seems to be more prone to ALS age-related alterations.

## 1. Introduction

The skeletal muscle is primarily responsible for human body support and movement [1]. It is particularly important in regulating whole-body metabolism and amino acid storage [1, 2]. It accounts for 40% of whole-body mass, though a progressive loss of muscle mass and strength (sarcopenia) is observed during aging [3–5]. This has been correlated with events of frailty and morbidity [6]. The loss of muscle mass is reflected by decreased size and number of type II (fast-twitch) fibers [7–9], which have been shown to have the ability of regulating body fat mass [2]. During aging, among other features, chronic oxidative stress leads to the accumulation of protein aggregates which have been widely characterized in several tissues [10, 11]. It is possible that these alterations are the reflection of disturbed proteostasis, which is characterized by an imbalance between anabolic and catabolic systems, promoting of cellular dysfunction. Within catabolic processes, cells possess two main proteolytic systems, the ubiquitin proteasomal system (UPS) and the autophagy-lysosomal system (ALS). While the UPS is responsible for the degradation of mildly oxidized proteins, by the 20S proteasome, as well as polyubiquitinated proteins, by the 26S proteasome [12, 13], the ALS has a wide variety of targets, which can range from proteins to whole organelles [14]. Briefly, the ALS can be divided mainly into two processes, the delivery of cytosolic constituents, so-called cargo, through autophagy *via* autophagosomes, and its degradation later on by the lysosomal hydrolases. The best described types of autophagy can be categorized into (i) chaperone-mediated autophagy, which consists in the recognition and unfolding of specific substrates that are directly translocated by heat-shock protein Hsc70 into the lysosomes *via* receptor lysosomal-associated membrane protein-2a (LAMP2a); (ii) microautophagy, which involves the invagination or engulfment of cytosolic proteins directly by lysosomes; and (iii) macroautophagy (hereafter referred as autophagy) [15, 16]. Autophagy is characterized by the elongation of a double membrane mainly from endoplasmic reticulum, but Golgi complex, mitochondria, and plasma membrane can also contribute to the donation of membrane material and together originate the what is called preautophagosome [17]. Further, autophagy regulatory proteins, such as the ATG5-ATG12 protein complex, are responsible for the expansion of the now called phagophore by stimulating the conjugation of the cytosolic form of microtubule-associated protein 1A/1B-light chain 3-I (LC3-I) with phosphatidylethanolamine (PE), leading to the formation of LC3-II [17, 18]. Another important protein for the determination of the autophagic process is the sequestosome1 (p62), an ubiquitin-binding protein. This protein can recognize ubiquitinated substrates with preferential affinity for K63-polyubiquitin chains. Further, it is incorporated together with LC3-II into the autophagosomes to be delivered and degraded by lysosomes, being considered reporters of autophagy activity [19–21]. Lysosomes contain different hydrolases, among others, cathepsins, mainly active in an acidic environment and responsible for the degradation of targeted protein cargo. Recently, we have shown that the UPS is negatively affected by aging in different skeletal muscles, favoring the accumulation of polyubiquitinated and oxidized proteins [22]. We thus hypothesized, based on current knowledge where a cross talk between the UPS and ALS has been described, that UPS inhibition might lead to ALS activation as a compensatory mechanism in different skeletal muscle types [12, 23]. Therefore, we aimed to evaluate and characterize the ALS in different skeletal muscle types. To meet that purpose, we extracted *musculus soleus* (SOL), mainly composed of slow-twitch fibers, and *musculus extensor digitorum longus* (EDL), consisting mostly of fast-twitch fibers, from young (4 months) and old (25 months) C57BL/6JRj mice. Afterwards, we characterized age-associated posttranslational protein modifications, such as of 3-nitrotyrosine (3-NT), pentosidine, and K63-polyubiquitinated proteins (K63). Moreover, to study the ALS, we analyzed gene and protein levels of autophagy- and lysosomal-related proteins, including ATG5-ATG12 complex, p62, LC3, and LAMP1, respectively. In addition, we determined changes in lysosomal activity, comparing cathepsin B and L activity in young and old SOL and EDL muscles. Interestingly, our results show and confirm that during aging, posttranslational protein modifications are increased in both muscles, more pronounced in old SOL. Furthermore, we were able to show that autophagy-related proteins were differently expressed in old SOL and EDL. Together, our results suggest that SOL is still able to compensate the already imbalanced proteostasis, while EDL, on the other hand, seems to no longer counteract the impaired autophagy-lysosomal degradation in aging.

## 2. Materials and Methods

### 2.1. Experimental Animals

C57BL/6JRj male mice, 5-6 young (4 months old) and 5-6 old (25 months old), were used. The animals were kept under 12 light/dark cycle at room temperature and fed *ad libitum* a standard chow. All animal procedures were performed in accordance with the guidelines of the German Law on the Protection of Animals and were approved by the local authorities (*Landesamt für Umwelt*, *Gesundheit und Verbraucherschutz*, Brandenburg, Germany).

### 2.2. Preparation of Tissue Extracts

The *musculus soleus* and *musculus extensor digitorum longus* were removed from euthanized mice (with isoflurane followed by cervical dislocation). The muscles were rapidly weighed, frozen in liquid nitrogen, and stored at -80°C until processed.

### 2.3. RNA Isolation and qPCR

Both muscles (~10 mg each) were homogenized with a tissue lyser (Qiagen) with 200 *μ*L of lysis buffer from Dynabeads mRNA DIRECT Kit (ThermoFisher Scientific no. 61012). The lysate was forced through a gauge needle in order to shear the DNA. Next, the lysate was incubated with Dynabeads Oligo (dT) and rotated at room temperature (RT) in order to hybridize polyA tail from mRNA with the oligo (dT) on the beads. The vials were placed on a magnet, and the supernatant was collected for protein quantification. Further protocol was followed according to the instructions of the manufacturer. Afterwards, 100 ng of mRNA was used for cDNA synthesis with SensiFAST cDNA Synthesis Kit (Bioline, no. BIO-65054). Quantitative real-time PCR reactions were performed in the presence of SYBR Green (Qiagen no. 330502) and DreamTaq™ Hot Start DNA Polymerase (ThermoFisher Scientific, no. 15619374). Primer sequences (forward and reverse) were used as follows: *Atg5*, 5′-AACTGAAAGAGAAGCAGAACCA-3′ and 5′-TGTCTCATAACCTTCTGAAAGTGC-3′; *Sqstm1* (p62), 5′-AGATGCCAGAATCGGAAGGG-3′ and 5′-GAGAGGGACTCAATCAGCCG-3′; *Map1lc3* (LC3), 5′-GACCAGCACCCCAGTAAGAT 3′ and 5′-TGGGACCAGAAACTTGGTCT-3′; *Rpl13a*, 5′-GTTCGGCTGAAGCCTACCAG-3′ and 5′-TTCCGTAACCTCAAGATCTGCT-3′; and *Actb* (actin beta), 5′-CACTGCCGCATCCTCTTCCT-3′ and 5′-GATTCCATACCCAAGAAGGAAGGC-3′ were used as internal normalization controls.

### 2.4. Immunoblotting

Protein quantification from the proteins in the supernatant, obtained from the mRNA isolation of the previous collected samples, was performed by Bradford. Therefore, proteins were precipitated by acetone overnight at -20°C, and lately centrifuged for 10 min at 14000 rpm. The excess of acetone was removed, and the pellet was let to dry. Further, reducing Laemmli buffer (25 mM Tris (pH 6.8), 50% glycerol, 4% SDS, and 0.08% bromophenol blue) was directly added to each sample. 10 *μ*g of protein was separated in sodium-dodecyl sulfate-polyacrylamide gel electrophoresis (SDS-page) and transferred to nitrocellulose membranes. Furthermore, membranes were blocked in Odyssey® blocking buffer (LI-COR Biosciences, No. 927-70001) for 1 h at RT, followed by incubation of primary antibodies overnight at 4°C. The following primary antibodies were used: mouse monoclonal ATG5-7C6 antibody (NanoTools no. 0262-100), mouse monoclonal p62 antibody (Abcam, ab56416), rabbit monoclonal LC3A/B (Cell Signaling no. 12741), rabbit monoclonal K63-linkage specific polyubiquitin (Cell Signaling no. 5621), rabbit polyclonal 3-nitrotyrosine (Abcam, ab110282), mouse monoclonal pentosidine (BioLogo, PEN012) and rabbit monoclonal LAMP1 (Cell Signaling no. 3243). Subsequently, membranes were probed with fluorescent-labeled secondary antibodies (IRDye 800CW or 680LT goat anti-rabbit IgG or anti-mouse IgG, LI-COR Biosciences). Immunodetection was performed by LI-COR Biosciences equipment from Odyssey®. Proteins were normalized to total protein amount *via* Ponceau S staining (0.1% Ponceau S and 5% trifluoroacetic acid).

### 2.5. Lysosomal Activity (Cathepsin B and L Activity)

Skeletal muscle samples (~10 mg each) were homogenized with a tissue lyser (Qiagen) in 300 *μ*L of 1 mM DTT/PBS and shaken in a thermomixer for 1 h at 4°C. Following incubation, lysates were sonicated on ice for 2 min at 50% amplitude and subsequently centrifuged for 20 min at 14000 rpm. Protein was quantified by Bradford assay. Further, 6 *μ*g of protein lysate was incubated with incubation buffer (containing 150 mM Na-acetate, 24 mM cysteine∗HCL, and 3 mM EDTA dihydrate) at pH 4.0 for 10 min. To measure the activity of the cysteine cathepsins B and L, the fluorogenic peptide Z-Phe-Arg-AMC (Enzo no. BML-p-139) was used as a substrate at a final concentration of 166 *μ*M. The resulting fluorescence, due to the cleavage and release of AMC, was measured in a black 96-well plate and monitored every minute for 2 h at 37°C. The excitation/emission was measured at 360/460 nm, respectively. The enzyme activity was calculated from standards of free 7-amino-4-methylcoumarin (AMC). The assay was controlled using a protease inhibitor cocktail, diluted accordingly to the instructions of the manufacturer (Sigma-Aldrich, P8340).

### 2.6. Statistical Analysis

Statistical analyses were performed using GraphPad Prism 8 software (GraphPad Software; San Diego, USA). Shapiro-Wilk test was used to determine the normal distribution of the tested variables. Statistical details for each experiment are provided in the figure legend. Statistically significant differences were considered at *p* ≤ 0.05.

## 3. Results

### 3.1. Aged Slow- and Fast-Twitch Muscles Display Higher Posttranslational Protein Modifications

**Figure 1:**
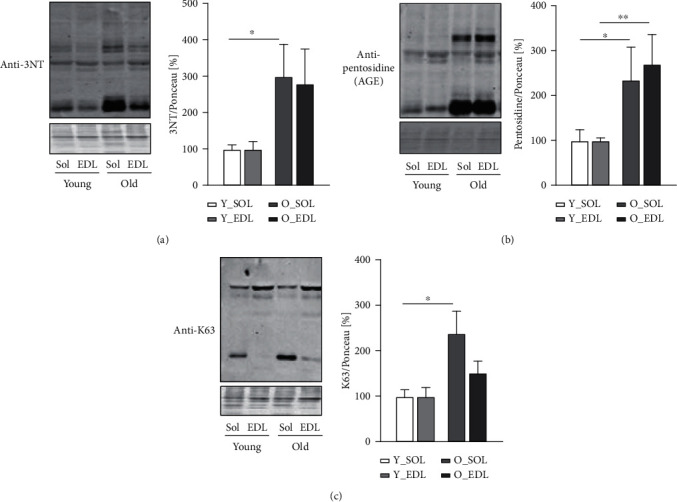
Posttranslational modifications in young and old SOL and EDL. All graphics show the comparisons between young SOL (white), young EDL (light grey), old SOL (grey), and old EDL (dark grey), by this order: (a) a representative immunoblot and quantification of 3-nitrotyrosine (3NT) protein against 3-nitrotyrosine (3NT) protein; (b) a representative immunoblot and quantification of pentosidine of pentosidine (AGE); (c) a representative immunoblot and quantification of K63-polyubiquitinated (K63) proteins. All blots with the respective densitometric quantifications are normalized with Ponceau S staining and further to the respective young muscle (results in percentage). For each analysis, 5-6 mice from each age group were used. Statistical significance was given as follows ^∗^*p* < 0.05 (Mann-Whitney test). Values are presented as mean ± s.e.m.

To assess alterations in proteostasis related to oxidative damage and the autophagy pathway, we initially quantified age-associated posttranslational protein modifications by immunoblot. We detected protein levels of 3-NT, a posttranslation modification, corresponding to the nitration of tyrosine residues due to increased oxidative and nitrosative stress (Figure 1(a)). Further, we analyzed protein levels of a biomarker of advanced glycation end products (AGEs), pentosidine, prone to form cross-linked aggregates (Figure 1(b)). Additionally, we evaluated K63 proteins, usually tagged to be degraded by autophagy-lysosomal system (Figure 1(c)). Densitometric quantification of 3-NT showed higher levels of tyrosine-nitrated proteins in both aged muscles, but only reaching significance in old SOL (Figure 1(a)). On the other hand, pentosidine was distinctly elevated in both aged SOL and EDL (Figure 1(b)). Furthermore, a significant increase in K63 proteins was observed in aged SOL muscle, but no changes in old EDL comparing to young EDL (Figure 1(c)).

### 3.2. Aged Slow- and Fast-Twitch Muscles Showed Differently Expressed Autophagy-Related Genes and Proteins

**Figure 2:**
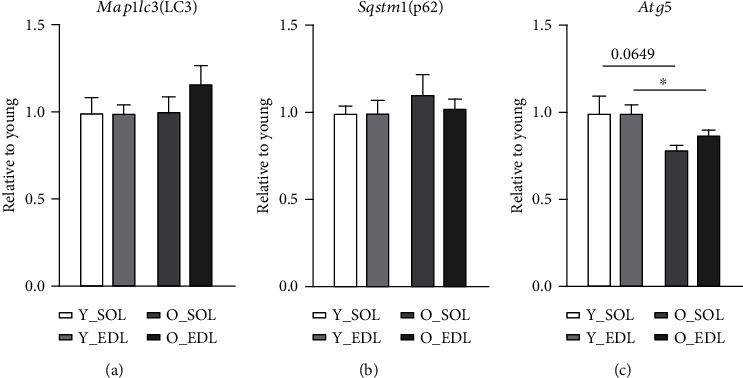
Autophagy-related genes during aging in SOL and EDL: (a) mRNA expression levels of *Map1lc3* (LC3), (b) *Sqstm1/p62*, and (c) *Atg5* in young and old slow (SOL) and fast-twitch muscle fibers (EDL). Six mice were used from each group. Statistical significance was given as follows ^∗^*p* < 0.05 (Mann-Whitney test). Values are presented as mean ± s.e.m.

**Figure 3:**
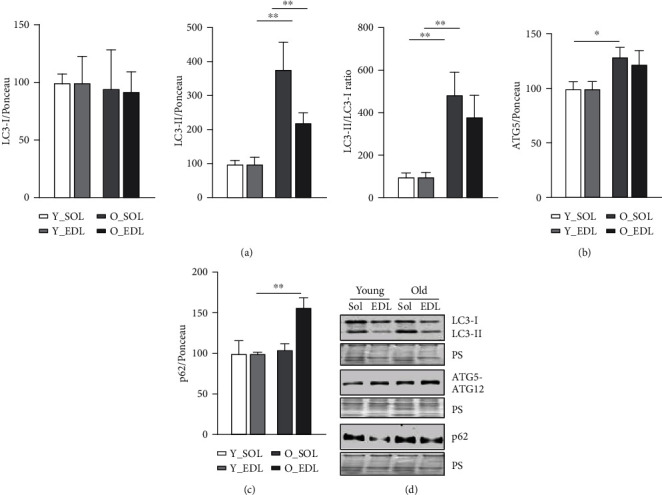
Autophagy-related protein levels during aging in SOL and EDL: (a) protein levels, from left to right, of LC3-I, LC3-II, and LC3-II/LC3-I ratio; (b) ATG5; (c) p62. Ponceau S staining was used for normalization in densitometric quantification, and further old groups were normalized to the respective young (results in percentage). (d) On the lower right, the figure depicts representative immunoblot images. 5-6 mice were used from each group. Statistical significance was given as follows: ^∗^*p* < 0.05 and ^∗∗^*p* < 0.01 (Mann-Whitney test). Values are presented as mean ± s.e.m.

Based on previous data that advanced the ALS as a compensatory mechanism upon UPS loss of function, we decided to investigate in detail the ALS age-related changes in both muscle types. To tackle this point, we initially assessed the transcription levels of key autophagy-related genes. The *Map1lc3* (LC3) and *Sqstm1/p62* (p62) mRNA levels (Figures 2(a) and 2(b), respectively) were not changed neither in aged SOL nor EDL. Nevertheless, *Atg5* gene expression level was significantly downregulated in old EDL (Figure 2(c)). We then analyzed protein levels, and the conversion of the cytosolic non-lipidated LC3-I into the autophagosomal membrane-associated protein (lipidated form) LC3-II. The latter was increased in both old SOL and EDL(Figure 3(a)). Therefore, the LC3-II/LC3-I ratio was significantly increased in both aged muscles (Figure 3(a)). The ATG5-ATG12 complex-containing proteins were significantly increased in old SOL and slightly higher in old EDL, yet not reaching statistical significance (Figure 3(b)). Interestingly, p62 protein, which can assemble misfolded polyubiquitinated proteins into aggregates (aggrephagy) [14], was increased in old EDL, while it remained unchanged in old SOL (Figure 3(c)).

### 3.3. Fast-Twitch Muscle Displays Higher Number of Lysosomes but Lower Lysosomal Activity

**Figure 4:**
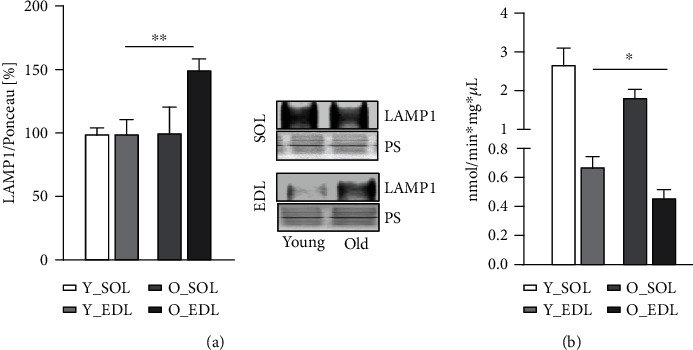
Fast-twitch muscle displays a higher number of lysosomes but lower lysosomal activity. (a) Immunoblot of LAMP1 protein in both young and old SOL and EDL muscles. Blots with the respective representative densitometric quantifications are normalized with Ponceau S staining (results in percentage). Statistical significance was given as follows: ^∗∗^*p* < 0.01 (Mann-Whitney test). (b) Lysosomal degradation after 2 h kinetics. Quantification from the kinetic release of AMC cleaved by cathepsins B and L from young and old muscles. Values expressed in nanomoles of product formed/minute^∗^milligram of protein. Muscle of 5 mice were used from each group. Statistical significance was given as follows ^∗^*p* < 0.05 (Student's *t*-test). For (a) and (b), values are presented as mean ± s.e.m.

While autophagy is likely to remain active in old SOL, the ALS appears to be negatively affected in old EDL. Interestingly, an increase in lysosomal number was found in old EDL, considering the observed significant increase in protein levels of lysosomal marker, LAMP1 (Figure 4(a)). To ascertain that the accumulation of modified proteins, and in the case of EDL, p62 is the result of impaired degradation, we measured cathepsin B and L activity as a proxy for lysosomal activity. In both SOL and EDL, cathepsin B and L activity was lower in aged muscles, comparing with younger counterparts (Figure 4(b)). Additionally, lysosomal activity of EDL in young animals was already lower than in SOL from young and old animals, suggesting a lower baseline level of activity in the EDL muscle. The assay was controlled with a protease inhibitor, which held a lower level of activity throughout the whole measurement (data not shown).

## 4. Discussion

Skeletal muscle holds a major role in regulating whole-body metabolism such as maintaining the core temperature by heat production and basal energy metabolism [1]. It also generates power and force by converting chemical into mechanical energy [1]. However, during aging, the skeletal muscle develops an altered structure and its functions are compromised [24], particularly in fast-twitch fibers (type II), which appear to be more prone to age-related changes [7]. These include the progressive accumulation of modified proteins and aggregates [11]. Several studies have shown that this phenomenon can be attributed to an impairment of the degradation systems, namely, the UPS and ALS [13]. However, there is ongoing controversy between different publications, on whether the proteolytic systems become up- or downregulated during aging [19, 25–28]. In our view, this could be explained by the metabolic and tissue variability between rodents and humans and also due to heterogeneous muscle fiber types used in the studies, since muscles that comprise both fiber types (fast and slow) provide more biological material for analyses and are easier to access. In our previous publication [22], we have characterized in detail how UPS, in subunit expression and enzymatic activity, differed in slow and fast muscle fiber types during aging, since both muscles display different metabolism. Moreover, we observed that both muscles declined in proteasomal activity. Thus, since we observed UPS impairment in both muscles during aging, we were interested in whether the ALS could be upregulated, as a compensatory mechanism accounting for proteasomal activity decrease. In this work, we set to understand how the ALS changes during aging in SOL and EDL, slow- and fast-twitch muscles, respectively. Therefore, we firstly characterized the amount of age-related protein modifications between young and old muscles in SOL and EDL, since muscles with different metabolism would probably exhibit a different content of protein modifications. Our results show an increased amount of 3-NT in old SOL (Figure 1(a)). This corroborates a study [29], where the authors found higher levels of 3-NT in quadriceps from aged rats compared to young counterparts. This was especially observed in mitochondrial fractions, suggesting that mitochondria can be the major source of oxidative stress. Similar results have been reported in other muscles from aged mice, such as in EDL [30] and in *M. gastrocnemius* [31]. 3-NT derives from the reaction of tyrosine and peroxynitrite, the latter a reactive nitrogen specie (RNS) (e.g., nitric oxide) which can be also produced in the mitochondria [32]. This could help to elucidate why SOL has higher 3-NT levels compared to EDL, particularly during aging, since it displays a higher mitochondrial and oxidative metabolism. Consequently, more ROS and RNS are produced. Nevertheless, there was also a higher amount of 3-NT in old EDL, yet it did not reach statistical significance. Among other posttranslational oxidative modifications, we investigated a product of glycosylation, pentosidine, known as advanced glycation end product and known to favor protein aggregation [33, 34]. Pentosidine was found to be significantly increased in both muscle fiber types in old mice (Figure 1(b)). Interestingly, this type of modification was also observed in old human *M. vastus lateralis* (~78 years old) when compared with young (~25 years old) [35]. Remarkably, this was also the case in serum of elderly patients and it was found to be correlated with loss of lean mass [33]. Another posttranslational protein modification that we detected was K63-polyubiquitination, a key event that directs proteins to autophagy. This particular type of ubiquitination process is characterized by the binding of one ubiquitin molecule to a lysine of the target substrate. K63-polyubiquitination involves a continuous linking of ubiquitin molecules to the lysine residue in position 63 of the previous linked ubiquitin. As the polyubiquitin chain forms, its structure works as a beacon to signal a protein for autophagy degradation. We found (Figure 1(c)) higher levels of K63-polyubiquitinated proteins in aged SOL, while no changes were observed in aged EDL. These posttranslational protein modifications all tend to increase in aged muscles, however, more prominently in old SOL than in old EDL. As explained above, one of the possible reasons might be a higher oxidative metabolism. This leads to the production of more reactive oxygen species and therefore having a higher demand for protein damage removal, comparing to old EDL (more glycolytic). In our view, this combined with the decline in proteasomal activity observed during aging [22] leads to an increment in the amount of proteins assigned to be degraded by ALS, thus reflecting K63-polyubiquitinated protein increase. To investigate whether autophagy could counteract the decline in proteasomal degradation and the accumulation of protein modifications, we assessed transcriptional and protein levels of autophagy-related proteins. We also found that *Map1lc3* (LC3) and *Sqstm1/p62* genes (Figures 2(a) and 2(b), respectively) remained unchanged between young and old SOL and EDL muscles, but the *Atg5* gene was significantly downregulated in old EDL (Figure 1(c)). At protein level, ATG5-ATG12 complex was significantly increased in old SOL but not changed in old EDL (Figure 3(b)). This complex is important for the lipidation process of LC3-II [36]. Raben et al. demonstrated, by means of muscle-specific *Atg5* inactivation in mice, an impairment of autophagy process, by the inhibition of LC3-II conversion [37]. In fact, the autophagy markers, LC3-II and LC3-II/LC3-I ratio, were remarkably increased in both aged muscles (Figure 3(a)), which can either indicate an increased autophagy flux, if further degraded [38], or an impairment of the ALS, leading to their accumulation. A remarkable and important difference to note is that, within both muscles and in parallel with increased LC3-II and LC3-II/LC3-I ratio levels of p62 protein were significantly higher in old EDL, which were not observed in old SOL. (Figure 3(c)). Together, the increase of these autophagy markers in old EDL suggests that the autophagy process is maintained. However, a later step of the ALS, such as fusion and/or degradation rate, is probably impaired, due to the accumulation of these proteins (Figures 3(a) and 3(c)), as we do not see changes in mRNA expression (Figures 2(a) and 2(b)). Interestingly, the scenario seems to differ in aged SOL, since we observe an increased LC3-II protein level as well as LC3-II/LC3-I ratio but no changes in p62 levels in parallel (Figure 3(a) and 3(c), respectively), which appears to be degraded, convincing us that the autophagy-lysosomal system remains functional. Our results seem to corroborate with previously published studies, where Penna et al. observed an increase in autophagy-related proteins, LC3-II and p62, in the *M. gastrocnemius* muscle from 24-month-old rats [27]. Wohlgemuth et al. also observed the increase LC3-II in *M. plantaris* from aged mice [28]. Contrariwise, LC3-I and LC3-II were found unchanged in the *vastus lateralis* muscle from elderly humans [38]. Furthermore, the amount of LAMP1 was significantly higher in old EDL, but not changed in old SOL (Figure 4(a)), which still does not justify the accumulation of autophagy-related proteins in old EDL. So far, since our results do not elucidate whether increased p62 protein levels in old EDL are due to impaired autophagy or defective lysosomal degradation, we decided to measure lysosomal activity. In fact, a slight reduction was observed in lysosomal activity in aged SOL, despite not reaching significance, while in aged EDL, it was remarkably declining when compared to younger muscle (Figure 4(b)). Nevertheless, the EDL lysosomal activity was already lower in younger age, compared to young SOL, as observed in Figure 4(b). However, young EDL also showed lower lysosomal number than young SOL (Figure 4(a)). In our view, the increased amount in lysosomes in old EDL during aging can be explained by a compensation of the cell, in order to recover the lower degradation and/or to reduce the increased amount of modified proteins [39, 40]. Interestingly, under disease conditions such as human cancer cachexia, lysosomal activity (cathepsins B and L) is increased [19], indicating that aging and catabolic conditions have distinct ways to counteract intracellular stress. Together, these results indicate that aged EDL seems more prone to a failure in ALS, evidenced by higher accumulation of autophagy and lysosomal markers and lower lysosomal activity, while old SOL still employs a moderate functional autophagy process, having a higher demand for removal of damaged proteins, due to oxidative metabolism, as also shown by Crupi et al. [41]. Together, and to our knowledge, these results reveal for the first time the differences between two metabolically different muscles, regarding the ALS. Here, we demonstrate how careful one should be when interpreting data from a whole different fiber-type composed muscle in terms of degradation processes, concretely, ALS, since different fiber types display different metabolism and ways to cope with intracellular stress. Besides these new insights, one has to be careful in judging the autophagy process in tissues, since it is hard to state which step of the autophagy might be affected. Therefore, *in vivo* animal studies with drug interventions would be advisable, as well as different muscle fiber-type isolation, in order to better understand the autophagy-lysosomal system flux during aging.

## 5. Conclusion

In summary, these results highlight the differences in the autophagy-lysosomal system between two different muscles, SOL and EDL, and show how differently they respond to intracellular stress, adjusted to their different metabolism. Nevertheless, more studies are required to fully understand the molecular mechanism of how the autophagy-lysosomal system affects muscle cell contractility and proteostasis in muscle tissues with different metabolism, to further develop strategies to preserve muscle function in aging.

## Data Availability

The data used to support the findings of the present study are available from the corresponding author upon request.
